# Post-intervention effects on screen behaviours and mediating effect of parental regulation: the HEalth In Adolescents study – a multi-component school-based randomized controlled trial

**DOI:** 10.1186/1471-2458-14-200

**Published:** 2014-02-25

**Authors:** Ingunn H Bergh, Maartje M van Stralen, Mona Bjelland, May Grydeland, Nanna Lien, Knut-Inge Klepp, Sigmund A Anderssen, Yngvar Ommundsen

**Affiliations:** 1 Department of Coaching and Psychology, Norwegian School of Sport Sciences, PB 4014 Ullevaal Stadion, Oslo NO-0886, Norway; 2 EMGO Institute for Health and Care Research and Department of Public and Occupational Health, VU University Medical Center, Amsterdam 1081 BT, the Netherlands; 3 Department of Nutrition, Faculty of Medicine, University of Oslo, Oslo NO-0316, Norway; 4 Department of Sport Medicine, Norwegian School of Sport Sciences, PB 4014 Ullevaal Stadion, Oslo NO-0886, Norway; 5 Department of Health Sciences and the EMGO Institute for Health and Care Research, Faculty of Earth and Life Sciences, VU University Amsterdam, de Boelelaan 1085, 1081, HV Amsterdam, the Netherlands

**Keywords:** Sedentary behaviour, Obesity prevention, Intervention, Mediation, Moderation, Adolescents

## Abstract

**Background:**

To improve effectiveness of future screen behaviour interventions, one needs to know whether an intervention works via the proposed mediating mechanisms and whether the intervention is equally effective among subgroups. Parental regulation is identified as a consistent correlate of screen behaviours, but prospective evidence as well as the mediation role of parental regulation is largely lacking. This study investigated post-intervention main effects on screen behaviours in the HEIA-intervention – a Norwegian school-based multiple-behaviour study, as well as mediation effects of parental regulation by adolescents’ and parents’ report. In addition, moderating effects of gender and weight status on the intervention and mediating effects were explored.

**Methods:**

Participating schools were randomized to control (n = 25) or intervention (n = 12) condition. Adolescents (n = 908 Control; 510 Intervention) self-reported their weekday and weekend TV-viewing and computer/game-use. Change in adolescents’ behaviours was targeted through school and parents. Adolescents, mothers (n = 591 Control; 244 Interventions) and fathers (n = 469 Control; 199 Intervention) reported parental regulation of the screen behaviours post-intervention (at 20 month). The product-of-coefficient test using linear regression analysis was conducted to examine main and mediating effects.

**Results:**

There was no intervention effect on the screen behaviours in the total sample. Gender moderated effect on weekend computer/game-use, while weight status moderated the effect on weekday TV-viewing and computer/game-use*.* Stratified analyses showed a small favourable intervention effect on weekday TV-viewing among the normal weight*.* Parental regulation did not mediate change in the screen behaviours. However, stronger parental regulation was associated with less TV-viewing and computer/game-use with effects being conditional on adolescents’ versus parental reports. Parental regulation of the screen behaviours, primarily by the parental report, was associated with change in the respective behaviours.

**Conclusion:**

Multiple behaviour intervention may not affect all equally well, and the effect may differ by weight status and gender. In future interventions parents should be encouraged to regulate their adolescents’ TV-viewing and computer/game-use on both weekdays and weekends as parental regulation was identified as a determinant of these screen behaviours*.* However, future intervention studies may need to search for more effective intervention strategies targeting parental regulation.

**Trial registration:**

Current Controlled Trials ISRCTN98552879

## Background

Overweight and obesity among children and adolescents are associated with adverse physical and psychosocial health consequences [1,2]. The high prevalence rates of both overweight/obesity and the unhealthy energy-balance related risk behaviour (diet, physical activity and sedentary behaviours) among young European adolescents calls for effective interventions [3]. Interventions targeting diet and physical activity have shown positive effects on BMI in children and adolescents [4]*,* and a recent meta-analysis show that reducing time spent on screen behaviours as a part of multiple behaviour interventions can affect BMI in young people [5]*.*

Overall sedentary time, as well as time spent in front of electronic screens such as TV and computers are associated with other unhealthy behaviours (e.g. snacking, smoking and alcohol use), poorer socio-cognitive development, unfavourable psychological and physical health outcomes, including higher body fatness in youth [6-10]*.* Adolescents engage in a variety of sedentary pursuits, but TV-viewing and computer-use (e.g. playing games, surfing on the internet, social media-use) contribute to a major portion of the time young people spend being sedentary [11,12]*.* In addition, screen behaviours are established quite early in life, and seem relatively stable over time [13,14].

Interventions to reduce screen behaviours have primarily focused on TV-viewing and have to a great extent been implemented as part of multiple behaviours interventions [9,15]. Meta-analyses show that interventions to reduce screen time can be effective, but effects are small [16,17]. The effect of recent multiple behaviour interventions on TV-viewing are however mixed, with some studies showing an effect [18], some revealing an unexpected effect [19] or studies showing an effect among subgroups only [20,21]*.*

Behaviours can be changed by interventions targeting modifiable factors associated with the behaviours, the so called mediators [22]. Knowledge about how to optimise effects of interventions targeting screen behaviour has been called for [16], but to improve effectiveness one needs to know whether an intervention works via the proposed mechanisms, by conducting mediation analysis [23].

Up till now most studies exploring mediators of intervention effects have focused on personal mediators [24]. Emerging evidence, however, shows that health behaviours in youth are highly influenced by environmental factors, and home environmental factors in particular [24]. Parental rules and regulation on screen time have been identified as one of few consistent modifiable correlates of children’s and adolescents’ screen behaviours [25-30], but prospective evidence of determinants of screen time behaviours is lacking [31]. Also, just a couple of studies have examined potential mediators of screen behaviour change ***,*** and none have to our knowledge investigated mediated effects of home environmental variables factors like parental regulation on changes in screen behaviours [24]. Furthermore, differences in associations of parents’ and adolescents’ report of regulation with adolescents’ TV-viewing and computer usage have been found [26], and investigation of both adolescents’ and parents’ report of screen behaviour regulation is warranted [32].

In addition, one type of intervention may not affect all equally well, and intervention effects and their mediators may not be equally effective across subgroups [33]. In the HEIA-study – a multi-component school-based obesity prevention intervention, we have previously reported mid-way effects (after eight months) on screen behaviours in girls but not in boys, while weight status moderated effect on computer/game-use among boys [34]*.* Hence, by examining whether intervention effects on screen behaviours are moderated by gender and weight status and whether gender and weight status moderate the mediators, it is possible to identify for whom an intervention is most (in)effective, as well as whether mechanisms of change differ by these subgroups.

The aims of the present study were: 1) to examine the effects of HEIA intervention on screen behaviours (TV/DVD-viewing and computer/game-use) post-intervention; 2) to assess whether parental regulation of TV-viewing and computer/game-use (as perceived by the adolescents and reported by mother and fathers) mediated intervention effects on screen behaviours and 3) to explore whether gender and weight status moderated the intervention and mediated effects.

## Methods

The HEIA study was a Norwegian 20 month randomized controlled school-based trial among 11-13 year-olds. A healthy weight development was promoted through targeting changes in physical activity, screen- and dietary behaviours. This paper focuses on screen behaviours only. The intervention intervened on determinants framed within a social-ecological approach as described in the conceptual model of the HEIA-study. A detailed description of the design and the development of the study is presented previously [35]*.*

Ethical approval and research clearance was obtained from the Regional Committees for Medical Research Ethics and the Norwegian Social Science Data Service.

### Procedure and participants

**Figure 1:**
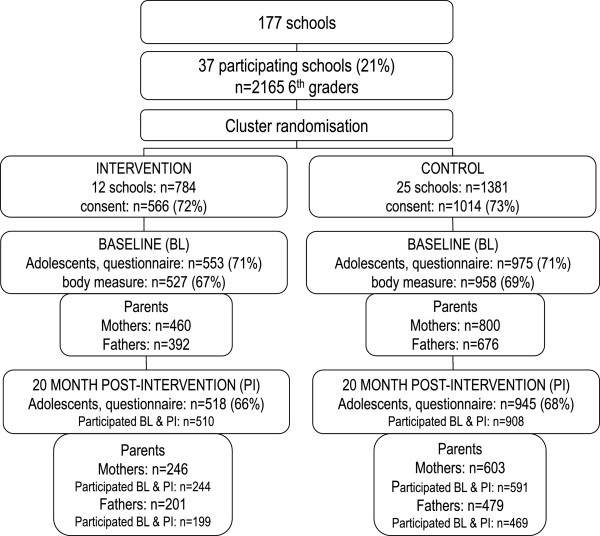
Flow diagram of recruitment, randomization, consent received and participants in the HEIA study.

Schools were recruited from town/municipalities in seven counties in the south-eastern part of Norway. For logistic reasons schools had to have at least 40 pupils enrolled in 6^th^ grade. Thirty seven schools out of 177 eligible schools accepted the invitation, and 12 schools were randomly assigned by simple drawing to the intervention group and 25 to the control group (Figure 1). All the 6^th^ graders in these schools (n = 2165) and their parents/legal guardians (hereafter called parents) were invited to participate. Of these, 1580 returned a signed, parental informed consent form for the adolescents.

Baseline data were collected during four weeks in September 2007 in 6^th^ grade and post-intervention data at the end of 7^th^ grade in May 2009. Adolescents (n = 1418: 908 Control; 510 Intervention) and their parents, both mothers (n = 835) and fathers (n = 668) who participated at both baseline and post-intervention are included in the analyses (Figure 1).

### Intervention

A 20 month multi-component intervention targeting energy-balance related behaviours was implemented in the two last years of primary school. It consisted of individual-, group-, and environmental strategies to promote a healthy development in the participants’ dietary, physical activity and screen time behaviours during school hours and in leisure time. The intervention was hypothesized to have a synergistic effect on the targeted behaviours, and is described in detail elsewhere [35,36].

The team of teachers at the involved grade levels led the implementation. Principals, school- and parent committees, and health nurses were also involved and informed about the intervention. The implementation was initiated by a yearly kick-off meeting with the teachers to ensure they knew the rationale and were familiar with the various intervention components. Throughout the school year the teachers received external support in the form of short monthly e-mail from the HEIA-study group to remind them about what parts of the intervention to implement.

The 6^th^ grade intervention targeted primarily the pupils’ diet and physical activity. In 7^th^ grade a computer-tailoring program was added to the intervention. The program included one session specifically targeting screen behaviours (plus one session targeting physical activity and two sessions targeting dietary behaviours). It was implemented during school hours and each session took about 15 minutes to complete. After completing the screen behaviour session in which the adolescents answered questions about their own screen behaviours (Additional file 1), each adolescent received a personal tailored feedback letter with specific suggestions on what (or not) and how to change their own TV-viewing and computer/game-use. In addition, two out of eight parental fact sheets implemented in 7^th^ grade included focus on screen behaviours. The first of these facts sheets informed parents about the targeted behaviours of the intervention in 7^th^ grade (including screen behaviours) and encouraged parental involvement. The theme for the other fact sheet was: “TV-viewing – the most common leisure time activity among Norwegian children/adolescents”. The specific targeted determinant in this fact sheet was parental regulation of TV-viewing and computer/game-use. This sheet was meant to be delivered to the parents by the adolescents after the completion of the screen behaviour computer tailoring session.

### Measures and procedures

The adolescents’ self-reported age, gender, potential mediators (parental regulation of TV-viewing and computer/game-use) and screen behaviours in an internet-based questionnaire which took about 45 min to complete, and participated in measurements of anthropometric parameters at baseline and post-intervention. Parental paper and pencil questionnaires were brought home to the parents by the adolescents and returned to the teachers in a sealed envelope which were collected from the school by project staff. Parental education was reported by the parents on the informed consent and categorized into 12 years or less, between 13 and 16 years and more than 16 years. The parent with the longest education was used for the adolescents’ parental educational background, or else the one available.

#### ***Adolescents’ screen behaviours***

Four questions with pre-coded answer categories were asked to assess usual TV-viewing (including DVD) and use of computer/electronic games: How many hours do you usually watch TV and/or DVD on a normal weekday? The same question was asked for a normal weekend day. The answer categories were (recoding in parentheses): half hour (0.5), one hour (1), two hours (2), three hours (3), four hours (4), five hours or more (5). The two questions on computer/electronic game-use were formulated in the same way as for TV/DVD, but the answer categories were: no playing (0), half hour or less (0.5), one hour (1), two hours (2), three hours (3), four hours or more (4).

#### ***Mediators***

The adolescents reported two mediators of screen behaviour both assessed by four items using a 5-point Likert scale (1 “totally disagree” to 5 “totally agree”) based on Hardy et al. [37]: *Perceived parental regulation of TV-viewing* (e.g. “My mother and father try to make sure I do not watch too much TV”; Cronbach’s alpha (α) at baseline/post-intervention of three items: 0.62/0.73) and *Perceived parental regulation of computer/game-use* (e.g. “My mother and father try to make sure that I do not use the computer and play games too much”; α at baseline/post-intervention of three items: 0.67/0.74).

The mothers and fathers also reported two (corresponding) potential mediators of the adolescents’ TV and computer/electronic game-use; *Parental regulation of TV-viewing* with six items using a 5-point Likert scale (e.g. “I permit my child to watch the TV programmes he/she wants to”; α at baseline/post-intervention of five items: 0.68/0.63 reported by mothers, 0.69/0.69 by fathers) and *Parental regulation of computer/game-use* with four items using a 5-point Likert scale (e.g. “I restrict how much time my child spend using the computer for playing games and so on”; α at baseline/post-intervention: 0.76/0.75 reported by mothers, 0.74/0.74 by fathers)*,* based on Hardy et al. [37]. Composite scores for each mediator variable were computed by summing the number of items divided by the numbers to keep the range across variables from 1.00-5.00.

A separate test-retest study was conducted prior to the main study; adolescents (n = 114), mothers (n = 44) and fathers (n = 35). The adolescents’ screen behaviours and perceived parental regulation of TV-viewing and computer/game-use, and parents reports of regulation of TV-viewing and computer/game-use showed either moderate, good or excellent test-retest results (ICC = 0.43-0.84).

#### ***Anthropometrics***

Height and weight of the adolescents were measured by project staff [35]. The baseline values were used to categorize the adolescents as normal weight and overweight/obese using the body mass index cut-offs values proposed by the International Obesity Task Force [38]*.*

### Statistical analyses

Independent t-tests and chi-square tests were conducted to test for differences between the intervention and control group in demographics, screen behaviours and mediators at baseline for adolescents and parents, and in the attrition analyses.

As school clustering effects explained only between 1.7-2.1% of the unexplained variance in the screen behaviours, all analyses were done without adjusting for the school clustering [39]. The product-of-coefficient test using linear regression was conducted to examine main and mediating effects [40] applying the script by Preacher & Hayes [41]*.* This test consists of: 1) estimating the main effect of the intervention on the four screen behaviours (c-coefficient); 2) estimating the effect of the intervention on changes in the potential mediators (a-coefficient); 3) estimating the independent effect of changes in the potential mediators on change in the screen behaviours adjusted for the intervention condition (b-coefficient); and (4) computing the product of the two coefficients (a*b), representing the mediated effect. All models were based on post-intervention variables adjusted for baseline values. The confidence interval of the mediated effect was calculated using bootstrapping with 1000 resamples of the data. Since mediating effects can still exist without a significant intervention effect on the outcome [42], mediation analyses were also conducted in its absence.

Secondly, in separate analyses the moderating influences of gender and weight status on the main effects were studied by linear regression models including the relevant interaction terms (e.g. intervention*gender). When significant moderating influences were revealed, subgroup analyses based on the moderator (girls vs. boys) and/or weight status (normal vs. overweight) were carried out for the main and mediated effects, using the same product-of-coefficient test applying the script by Preacher and Hayes [41].

**Figure 2:**
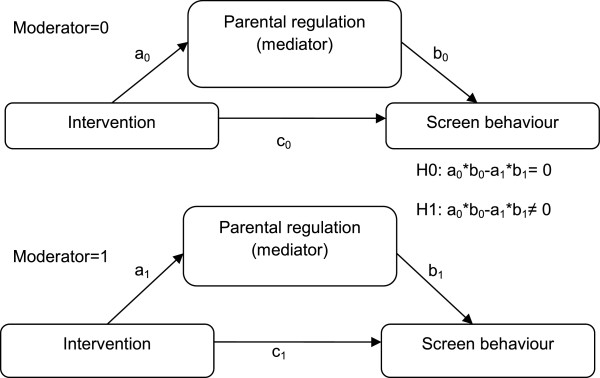
Conceptual model of moderation of a mediated effect.

Thirdly, the moderating influences of gender and/or weight status on the mediating effects were studied (Figure 2) by conducting the separate mediation model method. In this method, the ab-coefficients for the groups (normal weight vs. overweight) are compared (e.g. a*b_normal weight_ – a*b_overweight_) and if they were significantly different from each other, it indicates a significant moderation of the mediated effect. To test difference in ab-coefficients between the subgroups for statistical significance, the difference was divided by the pooled standard error (e.g. s_pooled_ = √(s^2^_ab_normal weight_ + s^2^_ab_overweight_) [43].

To control for potential effects of covariates, all analyses on TV-viewing were adjusted for parental education level and weight status, and all analyses on computer/game-use were adjusted for parental education level, weight status and gender. Analyses were performed using IBM SPSS Statistics, version 18.0. The alpha level was set at p < .05, except for the moderation test where p < .10 was used [33,44].

## Results

### Baseline characteristics

No significant baseline differences between the control and intervention group were found for the demographic variables or the screen behaviours (Tables 1 and 2).

**Table 1:** Baseline characteristics for intervention and control group in the HEIA study

	**Control**	**Intervention**	**p**
	**(n=908)**^**a**^	**(n=510)**^**a**^	
Age (mean; SD)	11.2 (0.27)	11.2 (0.26)	.38
**Gender**			
Girls (n; %)	434 (47.8%)	253 (49.6%)	.55
**Weight status**			
Overweight/obese (n; %)^b^	130 (14.5%)	55 (11.4%)	.12
**Parental education**			
Low (≤12 years) (n; %)	275 (31.1%)	129 (26.2%)	.15
13-16 years (n; %)	317 (35.8%)	186 (37.7%)	
>16 years (n; %)	293 (33.1%)	178 (36.1%)	

SD: standard deviation.

^a^n varies somewhat for weight status and parental education level.

^b^Overweight/obese is presented and treated as one group in the analyses due to the low proportion of obese (C=1.6%; I=1.2%).

**Table 2:** Descriptives for screen behaviours, main intervention effects in all and by weight status and gender

	**Baseline**^**a**^		**Post-intervention**			
	**Control (n = 908)**	**Intervention (n = 510)**	**Control (n = 908)**	**Intervention (n = 510)**	**Main intervention effect**	**p**
	**Mean (SD)**	**Mean (SD)**	**Mean (SD)**	**Mean (SD)**	**c-coeff (95% CI)**	
**Weekday TV-viewing**^**b,d**^						
All	1.45 (0.99)	1.47 (1.08)	1.70 (1.06)	1.63 (1.15)	-0.08 (-0.19, 0.04)	.18
Normal weight	1.39 (0.93)	1.48 (1.08)	1.66 (1.03)	1.58 (1.10)	-0.12 (-0.24, -0.01)	**.04**
Overweight	1.82 (1.21)	1.64 (1.15)	1.90 (1.16)	2.07 (1.39)	0.22 (-0.17, 0.62)	.26
**Weekend TV-viewing**^**e**^						
All	2.15 (1.19)	2.25 (1.29)	2.47 (1.23)	2.40 (1.27)	-0.12 (-0.25, 0.01)	.07
**Weekday comp/game-use**^**b,f**^						
All	1.13 (0.92)	1.07 (0.92)	1.37 (1.04)	1.32 (1.02)	-0.01 (-0.12, 0.11)	.93
Normal weight	1.09 (0.89)	1.05 (0.87)	1.36 (1.03)	1.28 (0.99)	-0.04 (-0.16, 0.08)	.49
Overweight	1.38 (1.02)	1.31 (1.16)	2.00 (1.21)	1.71 (1.20)	0.27 (-.0.10, 0.62)	.15
**Weekend comp/game-use**^**b,g**^						
All	1.51 (1.10)	1.53 (1.12)	1.82 (1.17)	1.78 (1.20)	-0.06 (-0.18, 0.06)	.36
Girls	1.24 (1.00)	1.20 (0.95)	1.68 (1.12)	1.51 (1.09)	-0.15 (-0.32, 0.03)	.09
Boys	1.75 (1.14)	1.84 (1.19)	1.94 (1.19)	2.05 (1.24)	0.02 (-0.16, 0.20)	.83

c-coeff: c-coefficient; Comp/game: computer/game-use; SD: standard deviation.

TV-viewing and comp/game-use reported as hours/day.

^a^Baseline differences for screen behaviours were tested with independent t-test; no significant differences were found.

^b^Subgroup analyses based on preceding significant interaction analyses by gender or weight status.

Effect analyses for the whole sample for TV-viewing were adjusted for weight status and parental education level.

Effect analyses for the whole sample for computer/game-use were adjusted for gender, weight status and parental education level.

n varies for the different behaviours:

^d^n in analyses for Weekday TV-viewing: All = 1299; normal weight =1130; overweight = 169.

^e^n in analyses for Weekend TV-viewing: All = 1281.

^f^n in analyses for Weekday comp/game-use: All = 1286; normal weight = 1119; overweight = 167.

^g^n in analyses for Weekend comp/game-use: All = 1272; girls = 618; boys = 654.

The attrition analyses showed no differences between adolescents who participated twice (n = 1418) and those participating at baseline only (n = 110) for baseline values for the screen behaviours, the mediators (parental regulation), the gender distribution, age or parental education. However, a higher number of overweight adolescents were found among those participating at baseline only (22.9% vs. 13.4%, p = 0.01). Among those lost to post-intervention, no differences between control and intervention group were found (data not shown).

In the subsample containing parents and adolescents (Table 3), TV-viewing weekday was significantly higher among those adolescents with mothers (1.5 vs. 1.4, p = .03) and fathers (1.5 vs. 1.4, p = .007) participating at baseline only (n mothers = 831; n fathers = 746) vs. both time-points (n mothers = 579, n fathers = 664), and computer/game-use weekday was significantly higher among those adolescents with fathers (1.2 vs. 1.0, p = 0.02) participating at baseline only (n = 744) vs. both time-points (n = 665) (data not shown).

**Table 3:** Baseline and post-intervention descriptives of parental regulation of TV-viewing and computer/game-use

	**Baseline**^**a**^		**Post-intervention**	
	**Control**	**Intervention**	**Control**	**Intervention**
	**Mean (SD)**	**Mean (SD)**	**Mean (SD)**	**Mean (SD)**
**Parental regulation TV-viewing, reported by**^**b,c**^				
Adolescents	3.64 (0.96)	3.68 (0.93)	3.41 (1.07)	3.38 (1.05)
Mothers	4.06 (0.75)	4.18 (0.66)*	3.92 (0.71)	4.02 (0.68)
Fathers	3.96 (0.75)	4.19 (0.71)***	3.80 (0.77)	3.90 (0.77)
**Parental regulation comp/game-use, reported by**^**b,c**^				
Adolescents	3.55 (0.99)	3.53 (1.01)	3.28 (1.11)	3.28 (1.10)
Mothers	4.13 (0.84)	4.20 (0.70)	3.98 (0.80)	3.97 (0.80)
Fathers	3.99 (0.82)	4.14 (0.72)*	3.77 (0.83)	3.87 (0.79)

*p <.05, ***p <.001, otherwise non-significant.

Comp/game: computer/game-use; SD: standard deviation.

^**a**^Baseline differences between control and intervention for mediators were tested with independent t-test.

^b^Range for regulation measures 1.00-5.00.

^c^n for parental regulation measures reported by: Adolescents 1390-1405; Mothers 764-775; Fathers 642-65.

### Intervention effect on screen behaviours

The results of the main effect analysis on the screen behaviours are shown in Table 2. There were no main effects seen in the total sample. However, weight status moderated the effect on weekdays TV-viewing (p = .04) and on computer/game-use (p = .08), and gender moderated the effect on weekend computer/game-use (p = .09). The following stratified analyses (Table 2) revealed an effect on weekday TV-viewing (c = -0.12; 95% CI (-0.24, -0.01); p = .04).

Table 3 shows the baseline and post-intervention values of the investigated mediators. Mothers’ and fathers’ report of regulation of TV-viewing and fathers’ report of regulation of computer/game-use were significantly higher in the intervention group, but baseline values were adjusted for in all analyses.

### Mediated effect

Table 4 shows effect of the intervention on the mediators (parental regulation), the effect of the mediator on the screen behaviours, and the mediated effects of all hypothesized mediators of the intervention effect on the screen behaviours reported by the adolescents, and the mothers and the fathers respectively in the total sample. The intervention did not affect parental regulation (a-coefficients) neither when reported by the adolescents themselves or by any of the parents.

**Table 4:** Intervention effect on mediators, effect of mediators on four screen behaviours and mediated effect

**Mediator: parental regulation of TV-viewing reported by**	**Effect on mediator a (95% CI)**	**Effect of mediator on weekday TV viewing b (95% CI)**	**Mediated effect ab (95% CI)**	**Effect on mediator a (95% CI)**	**Effect of mediator on weekend TV-viewing b (95% CI)**	**Mediated effect ab (95% CI)**
Adolescents^a^	-0.04 (-0.15; 0.07)	-0.04 (-0.10; 0.01)	0.00 (0.00; 0.01)	-0.03 (-0.14; 0.09)	-0.01 (-0.13; 0.00)	0.00 (0.00; 0.06)
Mothers^b^	0.03 (-0.07; 0.12)	-0.17 (-0.29; -0.06)**	-0.01 (-0.02; 0.01)	0.02 (-0.08; 0.12)	-0.16 (-0.30; -0.02)*	-0.00 (-0.02; 0.01)
Fathers^c^	-0.00 (-0.12; 0.11)	-0.04 (-0.15; 0.07)	-0.00 (-0.01; 0.01)	-0.02 (-0.14: 0.09)	-0.08 (-0.30; -0.02)	0.00 (-0.01; 0.03)
**Mediator: parental regulation of comp/game-use reported by**	**Effect on mediator a (95% CI)**	**Effect of mediator on weekday comp/game-use b (95% CI)**	**Mediated effect ab (95% CI)**	**Effect on mediator a (95% CI)**	**Effect of mediator on weekend comp/game-use b (95% CI)**	**Mediated effect ab (95% CI)**
Adolescents^a^	0.03 (-0.09; 0.15)	-0.01 (-0.06; 0.04)	-0.00 (-0.01; 0.00)	0.03 (-0.10; 0.15)	-0.02 (-0.07; 0.04)	-0.00 (-0.01; 0.00)
Mothers^b^	-0.04 (-0.15; 0.08)	-0.17 (-0.27; -0.07)***	0.07 (-0.01; 0.03)	-0.03 (-0.10; 0.15)	-0.05 (-0.16; 0.07)	0.00 (-0.00; 0.02)
Fathers^c^	0.05 (-0.08; 0.18)	-0.14 (-0.24; -0.04)**	-0.01 (-0.01; 0.03)	0.05(-0.10; 0.15)	-0.06 (-0.16; 0.07)	-0.00 (-0.03; 0.00)

*p < .05, **p < .01, ***p < .001, otherwise non-significant.

Comp/game-use: computer/game-use; SD: standard deviation.

Analyses for TV-viewing were adjusted for weight status and parental education level.

Analyses for computer/game-use were adjusted for gender, weight status and parental education level.

^a^n in analyses for adolescents: Weekday TV viewing = 1299; Weekend TV viewing = 1281; Weekday computer/game-use = 1286; Weekend computer/game-use = 1272.

^b^n in analyses for mothers: Weekday TV viewing = 708; Weekend TV viewing = 698; Weekday computer/game-use = 693; Weekend computer/game-use = 687.

^c^n in analyses for fathers: Weekday TV viewing = 601; Weekend TV viewing = 591; Weekday computer/game-use = 599; Weekend computer/game-use = 592.

Changes in adolescents’ perception of parental regulation of TV-viewing and computer/game-use were not associated with change in any of the corresponding screen behaviours. However, more regulation of TV-viewing by mothers’ reports was associated with less weekday TV-viewing (b = -0.17; (CI = -0.29, 0.06), p < .01) and weekend TV-viewing (b = -0.16; (CI = -0.30, -0.02); p < .05). Enhanced regulation of computer/game-use by both mothers’ (b = -0.17; (CI = -0.27, -0.07); p < .001) and fathers’ (b = -0.14; (CI = -0.24, -0.04); p < .01) reports were associated with less weekday computer/game-use. None of the hypothesized mediators reported by the adolescents or the parents mediated the intervention effect on any of the behaviours.

### Moderation of gender on mediated effect on weekend computer/game-use

The separate mediation models by gender for weekend computer/game-use (results not shown) did not reveal any intervention effects on change in parental regulation indices among girls or boys neither when reported by the adolescents nor by their parents, and parental regulation of computer/game-use was not associated with change in computer/game-use on weekends*.* No mediation effect in girls or boys or moderation effects of gender on the mediated intervention effects were found.

### Moderation of weight status on mediated effect on weekday TV and computer/game-use

Table 5 shows the separate mediation models by weight status for weekday TV-viewing and computer/game-use*.* There were no intervention effects on change in parental regulation indices among normal weight or overweight adolescents neither when reported by the adolescents nor their parents*.*

**Table 5:** Intervention effect on mediators, effect of mediators on outcomes, mediated effect and moderated mediation of weight status

**Mediators**	**Normal weight**			**Overweight**			
**Parental regulation of TV-viewing, reported by**	**Intervention effect on mediator a (95% CI)**	**Effect of mediator on weekday TV-viewing**^**a **^**b (95% CI)**	**Mediated effect ab (95% CI)**	**Intervention effect on mediator a (95% CI)**	**Effect of mediator on weekday TV-viewing**^**a **^**b (95% CI)**	**Mediated effect ab (95% CI)**	**Moderated mediation ∆ab (95% CI)**
Adolescents	- 0.05 (-0.18; 0.08)	-0.04 (-0.10; 0.02)	-0.02 (-0.00; 0.01)	0.00 (-0.34; 0.35)	-0.07 (-0.24; 0.10)	-0.00 (-0.06; 0.04)	0.01 (-0.01; 0.03)
Mothers	0.01 (-0.09; 0.10)	-0.13 (-0.26; -0.01)*	-0.00 (-0.02; 0.01)	0.22 (-0.13; 0.57)	-0.39 (-0.71; -0.06)*	-0.09 (-0.33; 0.01)	0.09 (0.08; 0.10)**
Fathers	-0.00 (-0.12; 0.12)	-0.07 (-0.18; 0.04)	-0.00 (-0.01; 0.02)	0.08 (-0.26; 0.42)	0.42 (-0.09; 0.93)	0.03 (-0.13; 0.29)	-003 (-0.09; 0.03)
**Parental regulation of comp/game-use, reported by**	**Intervention effect on mediator a (95% CI)**	**Effect of mediator on weekday comp/game-use**^**b **^**b (95% CI)**	**Mediated effect ab (95% CI)**	**Intervention effecton mediator a (95% CI)**	**Effect of mediator weekday comp/game-use**^**b **^**b (95% CI)**	**Mediated effect ab (95% CI)**	**Moderated mediation ∆ab (95% CI)**
Adolescents	0.05 (-0.08; 0.18)	-0.01 (-0.06; 0.05)	-0.00 (-0.01; 0.00)	-0.11 (-0.47; 0.24)	-0.04 (-0.19; 0.12)	0.01 (-0.01; 0.06)	-0.01 (-0.03; 0.05)
Mothers	-0.05 (-0.17; 0.07)	-0.15 (-0.26; -0.04)**	0.01 (-0.01; 0.04)	0.05 (-0.36; 0.47)	-0.28 (-0.55; -0.01)^#^	-0.02 (-0.16; 0.09)	0.03 (-0.07; 0.01)
Fathers	0.06 (-0.07; 0.20)	-0.16 (-0.26; -0.06)**	-0.01 (-0.04; 0.01)	-0.05 (-0.45; 0.36)	0.04 (-0.37; 0.45)	-0.00 (-0.14; 0.11)	-0.01 (-0.05; 0.03)

^#^p < .10, *p < .05, **p < .01, otherwise non-significant.

Comp/game-use: computer/game-use; SD: standard deviation.

Stratified analyses on TV-viewing were adjusted for parental education level.

Stratified analyses on computer/game-use were adjusted for gender and parental education level.

^a^n in analyses for Weekday TV-viewing: Adolescents: normal weight weight = 1130, overweight = 169; Mothers: normal weight = 620, overweight = 88; Fathers: normal weight = 538, overweight =63.

^b^n in analyses for Weekday comp/game-use: Adolescents: normal weight = 1119, overweight = 167; Mothers: normal weight = 607, overweight = 86; Fathers: normal weight = 535, overweight = 64.

Among the normal weights, increase in parental regulation of TV-viewing by mothers’ reports (b = -0.13, (CI = -0.26, -0.01); p < .05) was associated with less weekday TV-viewing. Increase in regulation of computer/game-use by both mothers’ (b = -0.15, (CI = -0.26, -0.04); p < .01) and fathers’ (b = -0.16; (CI = 0.26, -0.06); p < .01) reports was associated with less weekday computer/game-use.

Among the overweight adolescents, more regulation of TV-viewing by mothers’ reports (b = -0.39; (CI = -0.71, -0.06); p < .05) was associated with less weekday TV-viewing. No mediation effects were seen either among the normal weight or overweight adolescents. Still, weight status did moderate the mediating effect of mother’s regulation of weekday TV-viewing, indicating that mother’s regulation of TV-viewing in the intervention did differ between normal weight and overweight adolescents.

## Discussion

### Main and moderated effects on the screen behaviours

There was no main effect on any of the screen behaviours in the whole sample, but moderation effects of gender and weight status were revealed. The stratified follow-up analyses showed a significant effect among the normal weight (comprising 84% of the sample) on weekday TV-viewing. TV-viewing has been forwarded as the most important screen behaviour when it comes to prevention of overweight probably due to TV-viewings’ association with caloric intake (snacking) [45]. Nevertheless, the mid-way assessment (at 8 months) showed effect on both TV-viewing and computer/game-use, but in girls only [34]. In addition, the magnitude of the effect on daily weekday TV-viewing in this study (about 7 min/day) is less than the largest effect observed at the mid-way assessment (about 18 min/day) [34]. However, a reduction in effect as the intervention moves along is in line with the review by Kamath et al. [15] showing larger in-treatment effects than post-treatment effects of intervention targeting screen behaviours. Still, small effects for behaviours that are common in a large part of a population, as is the case for TV-viewing among youth, may be important at the population level [16].

The effect on TV-viewing may also have contributed to the favourable post-intervention effects observed in the HEIA-study for total sedentary time (22 min in girls) [46]. In addition, reduced TV-viewing among the normal weight could reflect that they have substituted parts of watching TV with physical activity. Indeed, a post-intervention effect on overall physical activity among the normal weight has been observed [46]. These effects taken together could contribute to a change in the energy-balance that might be of importance in obesity prevention. Also, a small effect on BMI has been observed among girls in the HEIA study [47]. However, both this study and the other findings from the HEIA-project, support previous research showing that energy-balance related behaviour interventions may not reach all equally well, and that the effect may vary by gender and weight status [4,33].

In addition, the intervention effect on weekday TV-viewing among the normal weight, together with the tendency (non-significant) for an effect in the undesired direction among the overweight (Table 2) may explain the apparent non-effect in the total sample. This is in line with the supposition by Kamath et al. [15] that variation in results could stem from studies with both normal weight and overweight compared to intervention with only normal weight. It is also discussed that prevention of excessive weight in those who are not yet overweight may be more effective than targeting mixed groups [48]. However, we can only speculate why the overweight did not respond to the intervention as intended. It might be that they lack alternatives to screen entertainment, or respond with a sort of reactance (by becoming more sedentary) when confronted with initiatives to reduce TV-viewing [49]*.* Indeed, unexpected results among the overweight group have also been seen previously in the HEIA-study. An unbeneficial effect on enjoyment of physical activity among the overweight has been reported [36]. At the same time there was no effect on accelerometer assessed physical activity among the overweight, but rather a tendency for an unfavourable effect in this group [46].

### Mediating effects and moderated mediating effects

In line with previous mediating studies of screen behaviours change, lack of mediated effects, were mainly due to the lack of intervention effect on the potential mediators [24]. Ineffective strategies or too low intervention dosage received may explain this. To provide parents with written material to the home may not be an effective strategy to reach parents [32]. In addition, the dose of the fact sheets might not have been powerful enough to create an impact on the potential mediators. Process evaluation also suggest that a relatively low proportion of the participating parents received all the fact sheets [50], and not all parents did respond to the parental questionnaire (Figure 1) which may indicate low parental involvement. In addition, the relatively high baseline values of the parental regulation constructs (mean: 3.53-4.20, Table 3) both by adolescents’ and parents’ reports may have led to ceiling effects, and it may also be that the measures were not sensitive enough to detect change. No mediation effect on screen behaviours have been identified by other previously examined potential mediators either [51-53]*.*

Although parental regulation was not affected by the intervention, changes in parental regulation of both TV-viewing and computer/game-use were associated with changes in the respective behaviours in the expected direction (b-coefficient analyses, Tables 4 and 5) with some differences among the subgroups. This is an important finding, providing prospective evidence for increase in parental regulation predicting reduction in screen behaviours.

Interestingly, mothers’ reports of regulation of screen behaviours were more often associated with reduction in the screen behaviours than fathers’ reports, which may mean that mothers are more involved in trying to regulate the adolescents’ TV-viewing and computer/game-use. However, both parents’ reports of regulation were more often found to be associated with change in the screen behaviours than adolescents’ perceived regulation of these behaviours. This difference may be due to young adolescents having difficulties recalling accurately or and/or parents being prone to a social desirability response bias. Another reason could be that parents and adolescents differ in their perception about parental practices. In addition, the wording of the regulation measures was a bit more specifically phrased to the parents compared to the adolescents, which could have influenced the results. However, it seems important to assess, include and describe reports by both parents (mothers and fathers) and children/adolescents, when assessing parental regulation of screen behaviours. In addition, both mothers and fathers should be encouraged to regulate their adolescents screen behaviours. Change in parental regulation was primarily found to be associated with change in weekday screen behaviours (except for mothers’ reports of regulation of weekend TV-viewing) which is in line with recent cross-sectional findings for restriction on sedentary behaviour [54]. Hence, interventions need to stimulate parents to regulate TV-viewing and computer/game-use on weekends also.

The intervention did not affect mother’s regulation of TV-viewing either among the normal weight or overweight adolescents, and no complete mediation effects were seen on change in weekday TV-viewing in either group. Still, we found a moderated mediating effect of weight status on mothers’ parental regulation on weekday TV-viewing (Table 5, ∆ab-coefficient). This result indicates that the mediating mechanisms of mothers’ parental regulation of TV-viewing weekday differed between normal weight and overweight adolescents. Stronger influence of mother’s regulation on TV-viewing among the overweight (Table 5, b-coefficient) may partly explain the result, which may also indicate that mothers’ parental regulation is a more influential determinant among overweight adolescents.

### Strength and limitations

This was a randomized controlled, long term intervention study including a relative large sample size with high retention rate for the adolescents. To our knowledge this is the first intervention study to investigate whether change in parental regulation, using both parents’ and adolescents’ reports, mediated young adolescents’ screen behaviours. Furthermore, main and mediated effects on both TV-viewing and computer/game-use were investigated, in addition to moderating effect of gender and weight status on the main effects on the screen behaviours and on the mediation mechanisms. Few studies have up to now included data from both mother and fathers, but this study explored mediation of parental regulation reported by both parents.

Both the internal consistency and the test-retest results (ICC) of the parental regulation measures were acceptable. However, the measures were phrased in a general format and did not differentiate between regulation on weekday and weekend as the report of screen behaviours did. A more context specific phrasing of the investigated mediators could possibly have improved their sensitivity to measure change. No differences for the screen behaviours were seen between those adolescents providing data at both time points and those lost to post-intervention. However, not all parents of participating adolescents did answer the parental survey, and the attrition analyses for the adolescents with parental data show that parents who did provide data at both time points were parents of adolescents having slightly more favourable screen behaviours than those adolescents with parents who were lost to post-intervention. So the parents included and retained in the study may be a biased subsample. Other limitations include the single items used to measure TV-viewing and computer/game-use, which gives only crude estimates [55]. However, the test-retest results (ICC) for these outcomes were moderate to high. While social desirability may have led to an under-reporting of the screen behaviours and a possible over-reporting of the potential mediators, descriptive results do not support this supposition given higher post-intervention values for the screen behaviours and lower post-intervention values of the mediators in both conditions (Tables 2 and 3). However, adolescents lost to post-intervention assessment were more likely to be overweight, which may indicate that the proportion of overweight may be somewhat lower in this sample than the population it represents. All the same, there were no differences between the control and intervention group among those lost to post-intervention or in the analysed sample.

## Conclusion

Taken together results revealed that this multiple behaviour intervention did not affect all equally well, by showing moderating effects by weight status and gender. Further studies should continue to investigate for whom interventions are effective or not. Parental regulation did not mediate screen behaviour effects and more intense targeting of this potential mediator may be needed probably combined with other strategies. Nevertheless, mothers’ and fathers’ reports of regulation of TV-viewing and computer/game-use seem to be social-environmental determinants of both TV-viewing and computer/game-use*.* Thus, parental regulation is warranted to target in future interventions, and both fathers and mothers should be encouraged to regulate their adolescents’ screen behaviours on both weekdays and weekends. However, more studies investigating mediation effect of screen behaviours are warranted.

## Competing interests

The authors declare that they have no competing interests.

## Authors’ contributions

IHB conducted the statistical analyses assisted by MM van S, wrote the first draft of the manuscript and made the greatest contribution to the paper. MB, MG, NL, K-IK, SAA and YO participated in designing the study, project planning and data collection. All authors have critically revised the manuscript, and read and approved the final version of the manuscript.

## Pre-publication history

The pre-publication history for this paper can be accessed here:

http://www.biomedcentral.com/1471-2458/14/200/prepub

## Supplementary Material

Additional file 1 Assessment of screen behavior time in the computer tailoring program. Click here for file
