# Supplementary material for: Post-intervention effects on screen behaviours and mediating effect of parental regulation: the HEalth In Adolescents study – a multi-component school-based randomized controlled trial
Source: BMC Public Health. 2014 Feb 25;14:200. doi: 10.1186/1471-2458-14-200 (PMC3946033; doi:10.1186/1471-2458-14-200)
Supplement: Additional file 1 — Assessment of screen behavior time in the computer tailoring program. [file 1471-2458-14-200-S1.pdf]

Assessment of screen behavior time in the computer tailoring program

Four questions were used to assess the adolescents' TV-viewing and computer/game-use in the computer tailoring program which was part of the 7th grade intervention:

1) How many days have you been watching TV/video/DVD's the last week?
2) How many days have you been using the computer for internet, chatting, e-mailing or for playing games (play-station, x-box) the last week?

The answer categories for both these questions were (recoding in brackets): not on any days [0], one day [1], two days [2], three days [3], four days [4], five days [5], six days [6], seven days [7].

3) On a regular day when you are watching TV, on average how long time are you watching?
4) On a regular day when you are using the computer, on average how long time are you using it?

The answer categories for both these questions were (recoding in brackets): less than 30 min [15], 30 min [30], 45 min [45], 1 hour [60], 1,5 hour [90], 2 hour [120], 2,5 hour [150], 3 hour or longer [180].

Based on the answers from these questions, the computer tailoring program calculated each participant's average daily screen behaviour time. In a personalized feedback letter the adolescents then received an advice on whether they ought to reduce their total daily TV-viewing and computer/game-use time when compared to the recommendation of no more than 2 hours pr day.
